# Systematic Review of the Pharmacological Evidence for the Selection of Antimicrobials in Bacterial Infections of the Central Nervous System in Dogs and Cats

**DOI:** 10.3389/fvets.2021.769588

**Published:** 2022-01-18

**Authors:** Robert Hertzsch, Angelika Richter

**Affiliations:** Faculty of Veterinary Medicine, Institute of Pharmacology, Pharmacy and Toxicology, Leipzig University, Leipzig, Germany

**Keywords:** antibiotics, cerebrospinal fluid, encephalitis, pharmacokinetic, meningitis

## Abstract

Bacterial meningitis in dogs and cats is a rare disease associated with a high lethality rate. The spectrum of causative bacteria includes a diverse set of gram positive, gram negative and anaerobic species. Currently, no veterinary medicinal product is approved for this indication in these species in Europe. The objective of this review was to collect the available pharmacokinetic data for antibiotics approved in dogs and cats to enable a preliminary analysis of their potential effectiveness for the treatment of bacterial meningitis. This analysis yielded data for 13 different antibiotics in dogs and two in cats. Additionally, data about frequently recommended cephalosporines not approved in dogs and cats were included. The collected data was used to assess the potential of the respective antibiotics to attain certain simple pharmacokinetic-pharmacodynamic (PK-PD) indexes in the cerebrospinal fluid (CSF). A more sophisticated investigation using modern methods was not possible due to the limited data available. For this purpose, data about the sensitivity of four bacterial species commonly associated with meningitis in dogs and cats to these antibiotics were included. The analysis provided evidence for the potential effectiveness of ampicillin, doxycycline, enrofloxacin, ceftriaxone and cefoxitin against bacteria frequently detected in bacterial meningitis in dogs. Data were not available or insufficient for the assessment of several antibiotics, including frequently recommended substances like metronidazole and trimethoprim-sulphonamide. Little evidence is available for the use of antibiotics in cats afflicted with this disease, highlighting the need for further research to obtain data for evidence based therapeutic recommendations.

## Introduction

Bacterial infections of the central nervous system (CNS) in dogs and cats can be caused by a diverse set of pathogens. Gram-positive bacteria like *Streptococcus* spp. and *Staphylococcus* spp. (1, 2), gram-negative bacteria like *Escherichia coli, Klebsiella* spp. and *Pasteurella* spp. (2, 3) as well as Mycobacteria (4) and anaerobic bacteria, such as *Bacteroides* spp. and *Fusobacterium* spp. (5), have been described as causative bacteria. Typical findings in cerebrospinal fluid (CSF) from animals with bacterial meningitis include abnormally high protein concentrations and pleocytosis (6). Intra- or extracellular bacteria may be detected by a thorough microscopic examination of CSF (7), which can be supported by Gram-stain analysis of the CSF (8). Even though the incidence of these infections in dogs and cats is unknown, a high lethality rate has been reported (2). As shown for bacterial meningitis in humans, prompt antibiotic therapy, preferably *via* intravenous injections, is mandatory to reduce the mortality rate to about 10% (9).

Antimicrobial agents should ideally be selected according to bacterial diagnosis to identify the pathogen and subsequent sensitivity testing (10). A precise identification of the species and strain of the causative pathogens requires a microbiological analysis of the sampled CSF. However, it can be difficult to obtain a proper diagnostic sample, i.e., lumbar CSF, in dogs and cats (11), and specific clinical breakpoints are not available (12). Furthermore, for the choice of antibacterial drugs for systemic treatment, knowledge of the pharmacokinetic properties, especially of their ability to cross the blood brain barrier (BBB), is required. Ideal antibiotics are small substances of low molecular weight, have a lipid-water partition coefficient of around 1–10, a volume of distribution of about 1 l/kg and low levels of plasma protein binding, properties which can differ within a group of antibiotics. In fact, the treatment of infections of the CNS is challenging because the penetration of antibiotics of the BBB and the blood cerebrospinal fluid barrier (BCSFB) is not only dependent on these properties of the antibiotic, but is also highly dependent on their affinity to transport systems and on the degree of meningeal inflammation (11–13).

The BBB is formed by endothelial tight junctions, basal lamina, pericytes, endfeet of astrocytes and perivascular microglia (13, 14). The endothelial cells exhibit a large number of drug transporters, such as P-glycoprotein (P-gp) and breast cancer resistance protein (15). With regard to the structure of the BCSFB and the different expression of transporters, the penetration of drugs into the interstitial fluid of the brain can differ from the penetration of the same drug into the CSF (16, 17). There is no gross diffusional barrier between the interstitial space of the nervous tissue and the CSF. Since direct measurement of unbound interstitial drug concentrations in the brain requires invasive microdialysis (18), CSF concentrations may be used as a surrogate method to predict brain interstitial fluid concentrations of drugs (19). Consequently, CSF is considered to be the medium of choice for clinical pharmacokinetic studies of drugs intended for the treatment of meningitis by the European Medicines Agency (EMA) (20). In addition, the steady state CSF to serum/plasma concentration ratio or the AUC_CSF_/AUC_Serum_ ratio is considered to be the best indicator of the penetration of a drug into the CNS (19). Based on a variety of underlying mechanisms, the permeability of the barriers for antibiotics can increase substantially during meningitis. Inflammatory cytokines like IL-6 and TNF-α can cause a decrease of the expression of molecules connecting the epithelial cells of the BBB and the BCSFB, such as occludin (21). Reactive oxygen species can also contribute to an increased permeability (22). Furthermore, the main efflux pumps of the BBB like P-gp are downregulated during inflammation of the meninges, leading to a slower clearance of substrate molecules from the CNS (23). These alterations during meningitis can lead to a substantial increase of the concentration of a drug in the CSF compared to healthy animals (24). Thereby, for instance minimal inhibitory concentrations (MIC) of aminopenicillins against *Streptococcus* spp. in CNS may be attained in patients suffering from meningitis. The duration of the increased permeability of the BBB and BCSFB after inflammation is not known in dogs and cats. A meningitis model which uses intracisternal lipopolysaccharide injections in rats demonstrated a return of the permeability of the BBB to baseline within 24 h after the injection (25). Therefore, it may be reasonable to assume that the function of the BBB is quickly restored after the subsiding of a CNS infection.

Because of the facts stated above, the selection of antibiotics that are able to sufficiently penetrate the CNS is critical. According to different databases, no veterinary medicinal product (VMP) is approved specifically for the treatment of bacterial CNS infections in cats and dogs in the European Union (26). VMPs approved for the indication meningitis are currently only available for pigs. The active substances used in these products are cefquinome, amoxicillin, benzylpenicillin-procaine in combination with dihydrostreptomycin and phenoxymethylpenicillin (26, 27). The selection of the optimal antibiotic is further complicated by the lack of clinical breakpoints for dogs and cats for this indication (12). Therefore, it is not known if a specific pathogen present in a patients CNS with a certain MIC can be considered as sensitive or resistant with regards to a certain antimicrobial substance based on antimicrobial susceptibility testing. In addition, there are no global guidelines for the treatment of bacterial infections of the CNS in dogs and cats.

In order to guide the rational selection of antibiotics for the treatment of bacterial meningitis in dogs and cats, the objective of the present study was to systemically collect all relevant available data about the CNS penetration of antibiotics which are approved for dogs and cats in the EU for various indications. Based on these data, the suitability of the investigated antimicrobial substances for the treatment of bacterial meningitis in dogs and cats was assessed.

## Materials and Methods

### Selection of Antibiotics Approved in Dogs and Cats

Lacking a comprehensive European Database of all VMPs which are approved in all European Union member states, we considered following databases to identify the antibiotic substances approved for the use in dogs and cats: (a) VETIDATA (26), including all VMPs approved *via* the central, the decentralized or national regulatory procedure in Austria and Germany, (b) the database of the Spanish Veterinary Medicines Authority (27), (c) the database of the Irish Veterinary Medicines Authority (28) and (d) the Heads of Medicines Agencies Database. Because of language barriers and varying usability of the 27 national VMP databases, it cannot be excluded that the list of antibiotic substances exclusively approved *via* the national procedure in a member state of the EU other than Austria, Germany, Ireland or Spain is missing from this list. In total, 36 different antibiotic substances approved in dogs and 34 different antibiotic substances approved in cats were identified (Table 1).

**Table 1:** List of antimicrobial substances approved for dogs and cats in the EU.

**Approved in dogs**	**Approved in cats**
Amoxicillin	Amoxicillin
Ampicillin	Ampicillin
Benzylpenicillin	Benzylpenicillin
Cefadroxil	Cefadroxil^*^
Cefalexin^*^	Cefalexin
Cefovecin	Cefovecin
Chloramphenicol	Chloramphenicol^*^
Chlortetracycline	Chlortetracycline^*^
Clindamycin	Clindamycin
Clavulanic Acid	Clavulanic Acid^*^
Cloxacillin	Cloxacillin
Colistin	Dihydrostreptomycin^*^
Dihydrostreptomycin^*^	Doxycycline
Doxycycline	Enrofloxacin^*^
Enrofloxacin	Fusidic acid^*^
Florfenicol^*^	Gentamicin
Fusidic acid	Kanamycin
Gentamicin	Lincomycin
Kanamycin	Marbofloxacin
Lincomycin	Metronidazole
Marbofloxacin	Neomycin
Metronidazole	Oxytetracycline
Neomycin^*^	Polymyxin B
Orbifloxacin	Pradofloxacin^*^
Oxytetracycline	Spectinomycin
Polymyxin B	Sulfadiazine
Pradofloxacin	Sulfadimethoxine^*^
Spectinomycin	Sulfadoxine^*^
Spiramycin^*^	Sulfamethazine/Sulfadimidine^*^
Sulfadiazine	Sulfamethoxypyridazine^*^
Sulfadimethoxine^*^	Sulfathiazole^*^
Sulfadoxine^*^	Thiostrepton^*^
Sulfamethazine/Sulfadimidine^*^	Trimethoprim
Thiostrepton^*^	Tylosin^*^
Trimethoprim	
Tylosin^*^	

* *For these compounds no records were identified by the ongoing database searching*.

### Study Selection: Inclusion Criteria

A comprehensive literature search was performed according to the Preferred Reporting Items for Systematic Reviews and Meta-Analyses (PRISMA) guidelines (29) for each identified antibiotic substance. The database PubMed (30) and the Web of Science Core Collection of the Web of Science database (31) were queried for publications entered into the databases before July 15^th^ of 2021. For each substance, the query with the structure “$substance cerebrospinal fluid $species” was performed. $substance stands for the respective substance, $species for dogs or cats. For example, studies about amoxicillin in dogs were searched for with the query “amoxicillin cerebrospinal fluid dogs.” The results of each query were screened for relevance based on their title and abstract. If warranted, the full article was assessed. No further constrains were used in the database search. In addition, references of relevant papers and broad database searches about pharmacokinetic data for the investigated active substances in dogs and cats were screened for potentially relevant publications.

An article was included into this study if all of the following inclusion criteria were met:

- The study was performed in dogs or cats.
- At least one of the active substances considered was investigated.
- The active substance was applied systemically.
- A direct measurement of the concentration of the investigated substance or its active metabolites was performed in the CSF or in the brain tissue.

**Figure 1:**
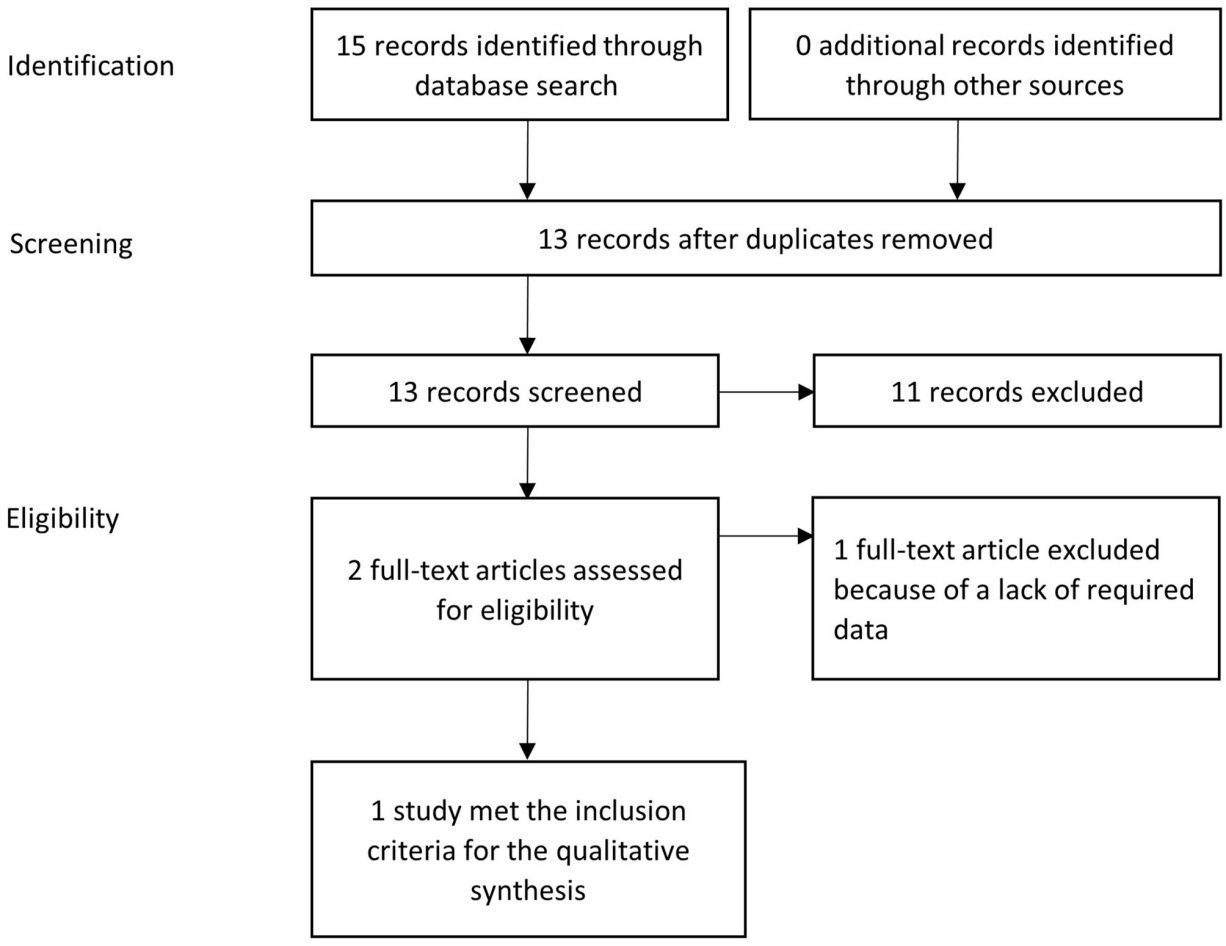
PRISMA flow diagram: systematic identification and selection of publications; example for ampicillin in dogs.

Figure 1 illustrates the PRISMA flow diagram used in the present study selection.

In addition to approved antibiotics, data on CSF concentration of cephalosporines in dogs were considered as these antimicrobial agents are often recommended for the treatment of bacterial meningitis in canine medicine (11).

### Exploratory Assessment of the Ability of Antibiotics to Achieve Effective CSF Concentrations

Literature data on CSF concentrations of various antibiotics in dogs and cats were compared to certain PK-PD indices. We considered a recent pan European study (32) on minimum inhibitory concentrations (MICs) of a variety of antimicrobial substances against bacterial pathogens relevant for bacterial meningitis in dogs and cats. Among those were *Staphylococcus aureus, Staphylococcus pseudintermedius, E. coli and Pasteurella multocida*, obtained from canine and feline patients with skin, wound and ear infections. If MIC data for a certain antibiotic or pathogen was not given in this study, supplemental data from other studies were included that investigated the aforementioned pathogens, namely *Staphylococcus pseudintermedius* (33, 34), *E. coli* (35) and *Pasteurella multocida* (36, 37). Unfortunately, we could not find recent European data for all pathogen–antibiotic combinations of interest in our study. Therefore, we also included data from other regions especially from North America. In addition, we considered the data on epidemiological cutoffs (ECOFF) from the EUCAST (38) database that includes data of human and animal origin. ECOFF data give relevant information about the susceptibility of wild type microorganisms without acquired resistance mechanisms against the respective antimicrobial. As no CLSI breakpoints for infections of the CNS in dogs and cats are available, the breakpoints specific for the respective species-antimicrobial-pathogen published for other tissues like skin of soft tissue were used. This approach enabled the calculation of the ratios C_max_ /MIC_50_, C_max_ /MIC_90_, C_max_/CLSI breakpoint (C_max_/BP) and C_max_/ECOFF.

## Results

### Selected Studies

The database search identified a total number of 124 potentially relevant studies for dogs and 69 potentially relevant studies for cats. After the addition of 8 studies from other sources in dogs and 3 in cats and the removal of multiple matches on the level of each respective substance, 114 hits for dogs and 66 hits for cats remained. Some investigated papers included data about multiple active substances. In this case, the investigation of each active substance was counted as one study. The screening of the title and abstract of all identified references reduced the number of studies that warranted a detailed assessment of their eligibility for the purpose of this paper to 19 in dogs and 7 in cats. In depth full text review of these papers reduced the number of studies included into the qualitative synthesis based on the inclusion criteria to 17 studies in dogs from 13 different papers and 3 studies from 3 different papers in cats. The main reasons for exclusion of a study at this point were a lack of data, the local application of an active substance or the use of toxic doses. A detailed list is given in Table 2 for dogs and in Table 3 for cats. Based on these studies, data on the pharmacokinetic properties of 13 different antibiotics with 22 specific dosage regimens in dogs and 2 antibiotics with 9 specific dosage regimens in cats were collected.

**Table 2:** Literature search: Number (n) of records (R) identified for approved antibiotics in dogs.

**Antibiotic**	**R from databases (n)**	**R from other sources (n)**	**R screened (n) after removal of duplicates**	**R excluded (n)**	**Full-text articles assessed for eligibility (n)**	**Full-text articles excluded, with reasons (n)**	**Studies included in qualitative synthesis (n)**
Amoxicillin	6	1	6	6	1	1	1
Ampicillin	15	0	13	11	2	1-lack of data	1
Benzylpenicillin	32	0	30	28	2	0	2
Cefadroxil	0	0	0	0	0	0	0
Cefovecin	1	0	1	1	0	0	0
Chloramphenicol	7	0	6	5	1	0	1
Chlortetra-cycline	0	1	1	0	1	0	1
Clavulanic acid	5	0	4	4	0	0	0
Clindamycin	7	0	5	5	0	0	0
Cloxacillin	1	0	1	1	0	0	0
Colistin	1	0	1	0	1	0	1
Doxycycline	10	1	8	7	1	0	1
Enrofloxacin	4	1	5	3	2	0	2
Fusidic acid	1	0	1	1	0	0	0
Gentamicin	4	0	4	2	2	0	2
Kanamycin	0	0	1	1	0	0	0
Lincomycin	2	0	2	2	0	0	0
Marbofloxacin	3	0	3	3	0	0	0
Metronidazole	3	1	3	2	1	1-lack of data	0
Orbifloxacin	2	0	2	2	0	0	0
Oxytetracycline	0	1	1	0	1	0	1
Polymyxin B	1	0	1	0	1	0	1
Pradofloxacin	1	2	3	1	2	0	2
Spectinomycin	2	0	1	1	0	0	0
Sulfadiazine	6	0	4	3	1	0	1
Trimethoprim	6	0	4	4	0	0	0
Sum	124	8	114	95	19	2	17

**Table 3:** Literature search: number (n) of records (R) identified for approved antibiotics in cats.

**Antibiotic**	**R from databases (n)**	**R from other sources (n)**	**R screened (n) after removal of duplicates**	**R excluded (n)**	**Full-text articles assessed for eligibility (n)**	**Full-text articles excluded, with reasons (n)**	**Studies included in qualitative synthesis (n)**
Amoxicillin	6	0	5	5	0	0	0
Ampicillin	7	0	7	7	0	0	0
Benzylpenicillin	19	1	20	17	3	2-1 toxicity test, 1 local application	1
Cefalexin	1	0	1	1	0	0	0
Cefovecin	0	1	1	0	1	1-no data	0
Chlor-amphenicol	7	0	7	7	0	0	0
Clindamycin	11	1	8	6	2	0	2
Cloxacillin	1	0	1	0	1	1-no data	0
Doxycycline	1	0	1	1	0	0	0
Gentamicin	1	0	1	1	0	0	0
Kanamycin	2	0	2	2	0	0	0
Lincomycin	2	0	2	2	0	0	0
Marbofloxacin	3	0	2	2	0	0	0
Metronidazole	1	0	1	1	0	0	0
Neomycin	1	0	1	1	0	0	0
Polymyxin	1	0	1	1	0	0	0
Spectinomycin	1	0	1	1	0	0	0
Sulfadiazine	3	0	3	3	0	0	0
Trimethoprim	1	0	1	1	0	0	0
Sum	69	3	66	59	7	4	3

### Pharmacokinetic Data in Dogs

Data about the pharmacokinetics of three different penicillins were collected (Table 4). For those substances, a higher penetration into the CSF was reported in dogs with meningitis compared to dogs with healthy meninges. Meningitis increased the ratio of the area under the curve (AUC) in plasma and CSF by the factor of 31 from 0.006 to 0.186 % for amoxicillin (24). A similar increase was reported in the literature for the mean peak concentration (C_max_) in CSF for ampicillin, which increased between 15 and 31 fold from 0.06 to 0.11 μg/ml to a level of 0.9 to 3.5 μg/ml in dogs with meningitis compared to dogs with healthy meninges (39). The free plasma fraction (fu) of aminopenicillins in dogs is high, ranging from 0.8 to 0.87 (52).

**Table 4:** Pharmacokinetic data of antibiotics in CSF of dogs.

**Antibiotic**	**CSF:serum-ratio (healthy meninges)**	**CSF:serum-ratio (meningitis)**	**Mean C_**max**_ [μg/ml] in CSF (healthy meninges)**	**Mean C_**max**_ [μg/ml] in CSF (meningitis)**	**fu**	**Dosage regimen**	**Ref**.
Amoxicillin	0.006 (AUC CSF/AUC plasma)	0.186 (AUC CSF/AUC plasma)	no data	no data	0.87	22 mg/kg i.v.	(24)
Ampicillin	no data	no data	0.06–0.11 μg/ml	0.9–3.5 μg/ml	0.8	25 mg/kg i.v.	(39)^*^
Ampicillin	no data	no data	0.4 μg/ml	no data	0.8	50 mg/kg i.v.	(39)^*^
Benzylpenicillin	no data	no data	0.2–0.3 μg/ml	1–15 μg/ml	0.4	20–30 mg/kg i.v.	(39)^*^
Benzylpenicillin	no data	no data	0.18 μg/ml	0.61 μg/ml	0.4	30.000 U/kg i.m.	(40)
Benzylpenicillin	no data	no data	0.28 μg/ml (1 h)	1.20 μg/ml (1 h)	0.4	30.000 U/kg i.v.	(40)
Benzylpenicillin	no data	no data	0.37 μg/ml	1.30 μg/ml	0.4	60.000 U/kg i.v. infusion over 4 h	(40)
Cefotaxime	0.036 (AUC CSF/AUC plasma)	0.163 (AUC CSF/AUC plasma)	no data	no data	0.4	50 mg/kg i.v.	(24)
Cefoxitin	0.008	0.083	1	10	n.d.	50 mg/kg i.v. infusion over 1 h	(41)
Cefoxitin	0.016	no data	2	no data	n.d.	100 mg/kg i.v. infusion over 1 h	(41)
Ceftriaxone	0.006 (AUC CSF/AUC plasma)	0.224 (AUC CSF/ AUC plasma)	0.41	15.2	0.75	50 mg/kg i.v.	(24)
Ceftriaxone	0.01 (AUC CSF/AUC plasma)	no data	1.56	no data	0.75	100 mg/kg i.v.	(24)
Chloramphenicol	0.24–18	no data	6.5 μg/ml	no data	0.7	50 mg/kg p.o.	(42)
Chlortetracycline	0.05–0.1 (plasma)	no data	0.26–0.5 μg/ml	no data	0.5^†^	10 mg/kg i.v.	(43)
Colistin	no data	no data	Not detected (LOD: 0.1 μg/ml)	no data	n.d.	4 mg/kg i.m. twice within 6 h	(44)
Doxycycline	0.2	no data	1 μg/ml	no data	0.0825	5 mg/kg i.v. bolus + 1 mg/kg per h i.v. infusion for 3 h	(45)^*^
Enrofloxacin	0.4 (graphic data)	no data	0.79 μg/ml	no data	0.65	5 mg/kg once daily (4 days p.o.-last day i.v.)	(46)^*^
Enrofloxacin	0.8	no data	5.3 μg/ml	no data	0.65	20 mg/kg i.v.	(47)
Gentamicin	0.058	0.113	0.7 μg/ml	0.9 μg/ml	0.9	4 mg/kg i.m.	(48)
Gentamicin	0.046	no data	1.,8 μg/ml	no data	0.9	6 mg/kg i.m.	(48)
Gentamicin	0.14	no data	1.3 μg/ml	no data	0.9	10 mg/kg i.v.	(49)
Oxytetracycline	<0.05	no data	<0.3 μg/ml	no data	0.75 ^†^	5 mg/kg i.v. bolus + 1 mg/kg i.v. infusion for 3 h	(45)^*^
Polymyxin B	no data	no data	Not detected (LOD: 0.,1 μg/ml)	no data	n.d.	4 mg/kg i.m. twice within 6 h	(44)
Pradofloxacin	0.35	no data	0.42 μg/ml	no data	0.64	3 mg/kg once daily for 5 days (day 1–4 p.o.; day 5 i.v.)	(46)^*^
Pradofloxacin	no data	no data	0.1 μg/ml	no data	0.64	6 mg/kg p.o. once daily for 6 days	(50)
Sulfadiazine	0.388 (AUC CSF/AUC plasma)	0.5 (AUC CSF/ AUC plasma)	12.5 μg/ml	14.5 μg/ml	0.85	12.5 mg/kg i.v. bolus + 1.25 mg/h i.v. infusion for 5 h	(51)^*^

* *Concentrations given as graphic data extracted via WebPlotDigitizer (https://apps.automeris.io/wpd/)*.

† *data from ruminants; n.d., no data.; fu, free plasma fraction*.

For benzylpenicillin, an increase of the mean C_max_ in CSF by a factor of 3.4 to 4.3 has been reported in dogs with meningitis compared to dogs with healthy meninges (40), corresponding to mean C_max_ of 0.18 to 0.37 μg/ml in CSF of healthy dogs and 0.61 to 1.3 μg/ml in dogs with meningitis, depending on the dose and time of sampling. Another study showed an even larger increase from 0.2 to 0.3 μg/ml CSF in healthy animals to a level of 1 to 15 μg/ml CSF in dogs with meningitis (39). The fu of benzylpenicillin in dogs is 0.4 (53).

For chloramphenicol, one study reported a CSF/plasma concentration ratio in healthy dogs of 0.24 to 18 in a time span of 1.5 to 12 h after a single oral application. The highest detected concentration of chloramphenicol in the CSF was 6.5 μg/ml 6 h after the application with a corresponding CSF/plasma concentration ratio of 0.77 (42). The fu of chloramphenicol is 0.7 (54).

As expectable from the properties of polypeptides, neither colistin nor polymyxin B reached CSF concentrations above the limit of detection (44).

Data for three different tetracyclines were found in the literature. For chlortetracycline, a CSF/plasma concentration ratio of 0.05 to 0.1 was detected 4 h after an intravenous injection, corresponding to a concentration of 0.26 to 0.5 μg/ml in CSF (43). For doxycycline, a CSF/serum concentration ratio of 0.2 was detected based on the absolute concentration of this active substance in both specimens and a mean concentration of 1 μg/ml in CSF 3 h after administration (45). In the same study, a mean concentration of <0.3 μg/ml oxytetracycline was detected in the CSF, corresponding to a CSF/serum ratio of < 0.05 based on the absolute concentration of oxytetracycline in both specimens. Doxycycline is highly bound to plasma proteins, its fu is 0.0825 (55). Data about the fu of Chlortetracycline and Oxytetracycline in dogs is not available, but data from ruminants indicates a larger unbound fractions ranging from 0.5 to 0.75 (56).

Gentamicin was the only aminoglycoside for which data were available. One study investigated the pharmacokinetics after intramuscular and intravenous application (48). In this study, the CSF/serum ratio was 0.058 in healthy dogs and 0.113 in dogs with bacterial meningitis. The maximum concentration of gentamicin in the CSF was 0.7 μg/ml in healthy animals dosed with 4 mg/kg and 1.8 μg/ml in animals that received 6 mg/kg. In dogs with bacterial meningitis, a concentration of 0.9 μg/ml was measured in the CSF. Gentamicin is largely unbound to plasma proteins with an fu of 0.9 (57).

For the group of fluoroquinolones, data about pharmacokinetic properties of enrofloxacin and pradofloxacin were found in the literature. The CSF/serum ratio of enrofloxacin was 0.4 with a corresponding maximum concentration of 0.79 μg/ml in CSF in one study (46) and a CSF/serum ratio of 0.8 with a corresponding maximum concentration in CSF of 5.3 μg/ml in a second study (47). For pradofloxacin, a CSF/serum concentration ratio of 0.35 with a corresponding maximum concentration in CSF of 0.42 μg/ml was reported in the literature (46). Another study with a different dosage and sampling regime detected a maximum concentration of 0.1 μg/ml in CSF (50). The unbound fractions of enrofloxacin and pradofloxacin are 0.65 and 0.64 respectively (58, 59).

Sulfadiazine was the only sulphonamide for which data were found in the literature. The reported CSF/serum ratio based on the respective AUC was 0.388 in healthy animals and 0.5 in dogs with bacterial meningitis. The maximum concentrations in CSF was 12.5 μg/ml in healthy and 14.5 μg/ml in diseased animals (51). Sulfadiazine is mostly unbound in canine plasma with a fu of 0.85 (60).

No data on cephalosporines approved in dogs and cats were available (Table 1). Since the cephalosporines cefoxitin, cefotaxime and ceftriaxone are frequently mentioned as treatment options in meningitis (52, 61, 62), we additionally considered studies on their CSF concentrations (Table 4). Ceftriaxone reaches relatively high concentrations in dogs with meningitis caused by *Staphylococcus aureus* (24). The study reported a C_max_ of 15.2 μg/ml in the CSF and a CSF AUC/plasma AUC ratio of 0.224 in the investigated dogs. In animals without meningitis, the C_max_ was only 0.41 μg/ml in the CSF, corresponding to a CSF AUC/plasma AUC ratio of about just 0.01. Similar CSF AUC/plasma AUC ratios were reported for the second-generation cephalosporine cefoxitin (about 0.01 in healthy animals and 0.185 in animals with meningitis) and the third-generation cephalosporine cefotaxime (about 0.036 in healthy animals and 0.16 in animals with meningitis), but no other data about these two substances were published in this paper. Concentration data for cefoxitin could be retrieved from another study (41), where a C_max_ of 1 and 10 μg/ml was achieved in the CSF of healthy dogs and dogs with meningitis, respectively. Data on protein binding was available for cefotaxime with and fu of 0.4 (63) and for cefoxitin with an fu of 0.75 (64).

### Pharmacokinetic Data in Cats

Data about the penetration of antibiotics in the CSF of cats could only be retrieved from the literature for clindamycin and benzylpenicillin in healthy cats (Table 5). Clindamycin exhibited a CSF/serum concentration ratio of 0.043 to 0.081 and a maximum concentration in CSF of 0.15 μg/ml to 0.44 μg/ml in healthy cats depending on the respective tested dosage and dosage regimen in one study (66). In contrast, another study was unable to detect clindamycin in the CSF of cats 6 h after the last of three consecutive administrations (67). The CSF/serum concentration ratio for penicillin G in healthy cats was 0.04 μg/ml 2 to 3 h after a single intramuscular injection, which corresponded to maximum concentrations in the CSF of 0.6 to 1.5 μg/ml, depending on the administered dose. At 7 h after a single injection, the CSF/serum ratio was 0.52 which was accompanied by a maximum concentration in the CSF of 0.78 μg/ml. The reported CSF/serum concentration ratio was 0.9 with a corresponding maximum concentration in the CSF of 0.6 μg/ml 7 h after the last of 5 consecutive administrations. The administration of a longer acting benzylpenicillin procaine formulation resulted in a CSF/serum concentration ratio of 0.2 and a maximum concentration in the CSF of 0.78 μg/ml 7 h after the last of 5 consecutive administrations (65). According to our knowledge, specific data about the plasma protein binding of clindamycin and benzylpenicillin in cats is not available.

**Table 5:** Pharmacokinetic data of antibiotics in CSF of cats.

**Antibiotic**	**CSF:serum-ratio (healthy meninges)**	**CSF:serum-ratio (meningitis)**	**C_**max**_ [μg/ml] in CSF (healthy meninges)**	**C_**max**_ reported in CSF (meningitis)**	**Dosage regimen**	**Reference**
Benzylpenicillin	0.04	no data	0.6	no data	15.000 IU /kg i.m	(65)
Benzylpenicillin	0.04	no data	1.5	no data	50.000 IU/kg i.m.	(65)
Benzylpenicillin	0.52	no data	0.78	no data	50.000 IU/kg i.m.	(65)
Benzylpenicillin	0.9	no data	0.6	no data	50.000 IU/kg i.m. every 12 h for 5 times -	(65)
Benzylpenicillin-Procain	0.2	no data	0.78	no data	50.000 IU/kg i.m. every 12 h for 5 times	(65)
Clindamycin	0.043	no data	0.15	no data	5.5 mg/kg p.o. bid for 10 days	(66)
Clindamycin	0.081	no data	0.44	no data	11 mg/kg p.o. bid for 10 days	(66)
Clindamycin	0.059	no data	0.38	no data	22 mg/kg p.o. daily for 10 days	(66)
Clindamycin	no data	no data	not detected (<0.01)	no data	12.5 mg/kg p.o. for 1.5 days (3 applications)	(67)

### Comparisons of CSF Concentrations in Dogs and Cats With MIC Data

Several different PK-PD indices do exist for the assessment of the efficacy of antimicrobials from the various classes. Due to the limited availability of data like the AUC, the half-life or information about the absorption or elimination rate with regards to the CSF, most PK-PD indices could not be applied in this paper. The only parameter consistently available was the C_max_. Therefore, we chose to use PK-PD indices and PK-PD cutoffs based on the C_max_. The limitations of this approach are discussed in more detail in the discussion.

The calculation of the ratios C_max_ /MIC_50_, C_max_ /MIC_90_, C_max_/CLSI breakpoint (C_max_/BP) and C_max_/ECOFF for the pathogens *Staphylococcus aureus, Staphylococcus pseudintermedius, E. coli and Pasteurella multocida* were performed to enable conclusions about the possible effectiveness of the investigated substances against infections of the CNS caused by these pathogens. Unfortunately, this was not possible for all substances which are commonly used for the treatment of meningitis in dogs and cats because of a lack of data. The calculated ratios were compared to Pk-PD cutoffs for the respective substance. The results of this analysis are summarized in Table 6 for dogs and Table 7 for cats. The attainment of a PK-PD cutoff was deemed possible if the given parameter like the MIC_50_ or MIC_90_ was below the free concentration where the C_max_/MIC ratio linked to a possible antimicrobial effectiveness was still met. For instance, gentamicin for which a C_max_ of 1.8 μg/ml CSF has been reported in dogs, the PK-PD cutoff used by us expressed *via* the C_max_/MIC ratio is 10 (68). Consequently, the PK-PD cutoff used in our study is 1.8 μg/ml divided by 10 which equals 0.18 μg/ml. This means, that gentamicin would be potentially effective against a pathogen with a MIC_50_ or MIC_90_ lower than 0.18 μg/ml and most likely less effective or not effective against a pathogen with a MIC_50_ or MIC_90_ higher than 0.18 μg/ml.

**Table 6:** PK-PD-Target attainment for various antibiotics and different pathogens in dogs.

**Antibiotic/Pathogen**	***Staphylococcus pseudintermedius***				***Staphylococcus aureus***			

	**C_**max**_ /MIC_**50**_**	**C_**max**_/ MIC_**90**_**	**C_**max**_ /BP**	**C_**max**_/ ECOFF**	**C_**max**_/ MIC_**50**_**	**C_**max**_/ MIC_**90**_**	**C_**max**_/ BP**	**C_**max**_/ ECOFF**
Ampicillin	+	-	-	0	-	-	0	0
Ampicillin (m.)	+	-	+	0	+	-	0	0
Benzylpenicillin	+	-	0	+	-	-	0	+
Benzylpenicillin (m.)	+	-	0	+	+	-	0	+
Cefoxitin	0	0	0	0	-	-	0	-
Cefoxitin (m.)	0	0	0	0	0	0	0	+
Ceftriaxone	0	0	0	0	-	-	0	-
Ceftriaxone (m.)	0	0	0	0	+	+	0	-
Chloramphenicol	-	-	0	0	-	-	0	-
Chlortetracycline	-	-	0	0	0	0	0	0
Doxycycline	+	-	+	+	0	0	0	-
Enrofloxacin	+	-	+	+	+	-	+	0
Gentamicin	+	-	0	-	-	-	0	-
Oxytetracycline	-	-	0	0	0	0	0	0
Pradofloxacin	-	-	-	0	-	-	0	0
**Antibiotic/Pathogen**	***E. coli***				***P. multocida***			
Ampicillin	-	-	-	-	+	+	0	-
Ampicillin (m.)	-	-	+	-	+	+	0	+
Benzylpenicillin	-	-	0	0	+	+	0	-
Benzylpenicillin (m.)	-	-	0	0	+	+	0	+
Cefoxitin	-	-	0	-	0	0	0	0
Cefoxitin (m.)	+	+	0	-	0	0	0	0
Ceftriaxone	0	0	0	+	0	0	0	0
Ceftriaxone (m.)	0	0	0	+	0	0	0	0
Chloramphenicol	-	-	0	-	+	+	0	+
Chlortetracycline	0	0	0	0	0	0	0	0
Doxycycline	0	0	0	-	0	0	0	-
Enrofloxacin	+	-	+	+	+	+	0	+
Gentamicin	-	-	-	-	-	-	0	-
Oxytetracycline	0	0	0	0	0	0	0	0
Pradofloxacin	+	-	-	0	+	+	0	0

“*+” indicates possible attainment of the respective PK-PD-target,”-“ indicates target attainment is unlikely, “0” = lack of data; m.=meningitis, Specific data on MICs, ECOFFs and breakpoints (BP) used are given in Tables 8–11*.

**Table 7:** PK-PD-Target attainment for various antibiotics and different pathogens in cats.

	***Staphylococcus pseudintermedius***		***Staphylococcus aureus***		***E. coli***		***Pasteurella multocida***	

**Antibiotic**	**C_**max**_ / MIC_**50**_**	**C_**max**_ /MIC_**90**_**	**C_**max**_ / MIC_**50**_**	**C_**max**_ /MIC_**90**_**	**C_**max**_ / MIC_**50**_**	**C_**max**_ /MIC_**90**_**	**C_**max**_/MIC_**50**_**	**C_**max**_ /MIC_**90**_**
Benzylpenicillin	+	+	+	-	-	-	+	+
Clindamycin	+	-	+	-	0	0	-	-

*For explanations see legend to Table 6. Specific data on MICs and breakpoints (BP) used are given in **Table 12***.

**Table 8:** C_max_ in CSF in dogs after the administration of various antibiotics in healthy dogs or during meningitis (ampicillin, benzylpenicillin) and selected PK-PD cutoffs of *Staphylococcus pseudintermedius*.

**Antibiotic**	**C_**max**_ (μg/ml)**	**MIC_**50**_ (μg/ml)**	**MIC_**90**_ (μg/ml)**	**EUCAST ECOFF (μg/ml)**	**CLSI breakpoint for soft tissue infections (μg/ml)**	**PK-PD cutoff in μg/ml**	**Source of MIC**
Ampicillin (meningitis)	3.5	0.12	2	no data	0.25	1.75	32
Ampicillin	0.40	0.12	2	no data	0.25	0.2	32
Benzylpenicillin (meningitis)	1.30	0.06	4	0.03	no data	0.65	32
Benzylpenicillin	0.37	0.06	4	0.03	no data	0.185	32
Chloramphenicol	6.5	4	16	no data	no data	3.25	32
Chlortetracycline	0.5	0.5	16	no data	no data	0.17	33
Doxycycline	1	0.063	4	0.125	0.12	0.33	34
Enrofloxacin	5.3	0.12	1	0.5	0.5	0.53	32
Gentamicin	1.8	0.12	8	0.25	no data	0.18	32
Oxytetracycline	0.3	0.5	16	no data	no data	0.1	33
Pradofloxacin	0.42	0.06	0.25	no data	0.25	0.042	32

**Table 9:** C_max_ in CSF in dogs after the administration of various antibiotics in healthy dogs or during meningitis (ampicillin, benzylpenicillin) and selected PK-PD cutoffs of *Staphylococcus aureus*.

**Active substance**	**C_**max**_ (μg/ml)**	**MIC_**50**_ (μg/ml)**	**MIC_**90**_ (μg/ml)**	**EUCAST ECOFF (μg/ml)**	**CLSI breakpoint for soft tissue infections (μg/ml)**	**PK-PD cutoff in μg/ml**	**Source of MIC**
Ampicillin (meningitis)	3.5	1	16	no data	no data	1.75	32
Ampicillin	0.40	1	16	no data	no data	0.2	32
Benzylpenicillin (meningitis)	1.30	0.5	8	0.125	no data	0.65	32
Benzylpenicillin	0.37	0.5	8	0.125	no data	0.185	32
Cefoxitin	1	no data	no data	4	no data	0.5	no data
Cefoxitin (meningitis)	10	no data	no data	4	no data	5	no data
Ceftriaxone	0.41	no data	no data	8	no data	0.025	no data
Ceftriaxone (meningitis)	15.2	no data	no data	8	no data	7.6	no data
Chloramphenicol	6.5	8	8	16	no data	3.25	32
Chlortetracycline	0.5	no data	no data	no data	no data	0.17	no data
Doxycycline	1	no data	no data	0.5	no data	0.33	no data
Enrofloxacin	5.3	0.12	8	no data	0.5	0.53	32
Gentamicin	1.8	0.25	0.5	2	no data	0.18	32
Oxytetracycline	0.3	no data	no data	no data	no data	0.1	no data
Pradofloxacin	0.42	0.12	4	no data	no data	0.042	32

**Table 10:** C_max_ in CSF in dogs after the administration of various antibiotics in healthy dogs or during meningitis (ampicillin, benzylpenicillin) and selected PK-PD cutoffs of *E. coli*.

**Active substance**	**C_**max**_ (μg/ml)**	**MIC_**50**_ (μg/ml)**	**MIC_**90**_ (μg/ml)**	**EUCAST ECOFF (μg/ml)**	**CLSI breakpoint for soft tissue infections (μg/ml)**	**PK-PD cutoff in μg/ml**	**Source of MIC**
Ampicillin (meningitis)	3.5	4	32	8	0.25	1.75	32
Ampicillin	0.40	4	32	8	0.25	0.2	32
Benzylpenicillin (meningitis)	1.30	4	256	no data	no data	0.65	35
Benzylpenicillin	0.37	4	256	no data	no data	0.185	35
Cefoxitin	1	1	4	8	no data	0.5	35
Cefoxitin (meningitis)	10	2	4	8	no data	5	35
Ceftriaxone	0.41	no data	no data	0.125	no data	0.025	no data
Ceftriaxone (meningitis)	15.2	no data	no data	0.125	no data	7.6	no data
Chloramphenicol	6.5	8	16	16	no data	3.25	32
Chlortetracycline	0.5	no data	no data	no data	no data	0.17	no data
Doxycycline	1	no data	No data	4	no data	0.33	no data
Enrofloxacin	5.3	0.03	8	0.125	0.5	0.53	32
Gentamicin	1.8	1	2	2	2	0.18	32
Oxytetracycline	0.3	no data	no data	no data	no data	0.1	no data
Pradofloxacin	0.42	0.015	4	no data	0.25	0.042	32

**Table 11:** C_max_ in CSF in dogs after the administration of various antibiotics in healthy dogs or during meningitis (ampicillin, benzylpenicillin) and selected PK-PD cutoffs of *Pasteurella multocida*.

**Active substance**	**C_**max**_ (μg/ml)**	**MIC_**50**_ (μg/ml)**	**MIC_**90**_ (μg/ml)**	**EUCAST ECOFF (μg/ml)**	**CLSI breakpoint for soft tissue infections (μg/ml)**	**PK-PD cutoff in μg/ml**	**Source of MIC**
Ampicillin (meningitis)	3.5	0.12	0.12	0.5	no data	1.75	37
Ampicillin	0.40	0.12	0.12	0.5	no data	0.2	37
Benzylpenicillin (meningitis)	1.30	0.06	0.12	0.5	no data	0.65	37
Benzylpenicillin	0.37	0.06	0.12	0.5	no data	0.185	37
Chloramphenicol	6.5	0.25	0.5	1	no data	3.25	37
Chlortetracycline	0.5	no data	no data	no data	no data	0.17	no data
Doxycycline	1	no data	no data	1	no data	0.33	no data
Enrofloxacin	5.3	0.015	0.06	0.06	no data	0.53	37
Gentamicin	1.8	4	4	8	no data	0.18	37
Oxytetracycline	0.3	no data	no data	no data	no data	0.1	no data
Pradofloxacin	0.42	0.008	0.015	no data	no data	0.042	36

The analysis of the data for dogs presented in Table 6 shows that the PK-PD-targets for the index **C**_**max**_**/MIC**_**90**_ could not be met by any of the listed active substances for *Staphylococcus pseudintermedius*, whereas these targets were met by cefoxitin and ceftriaxone during conditions encountered in meningitis for *Staphylococcus aureus*. Cefoxitin was the only antibiotic that met this target for *E. coli* during meningitis. For *Pasteurella multocida*, chloramphenicol, enrofloxacin and pradofloxacin met their respective targets. The PK-PD target for the index **C**_**max**_**/MIC**_**50**_ was met by five active substances for *Pasteurella multocida*, by five active substances for *Staphylococcus pseudintermedius*, by five active substances for *Staphylococcus aureus* and three active substances, i.e., enrofloxacin, pradofloxacin and cefoxitin during meningitis for *E. coli*. The PK-PD target for the index **C**_**max**_**/BP** could only be computed for a small number of drug-pathogen combinations because of the limited number of available breakpoints. Ampicillin met this target for *Staphylococcus pseudintermedius* and *E. coli* but only with concentrations achieved during meningitis. Doxycycline met this target for *Staphylococcus pseudintermedius*. Enrofloxacin met this target for *Staphylococcus pseudintermedius, Staphylococcus aureus* and *E. coli*. The PK-PD target for the index **C**_**max**_**/ECOFF** was met by benzylpenicillin, doxycycline and enrofloxacin for *Staphylococcus pseudintermedius* and by benzylpenicillin for *Staphylococcus aureus*. For *E. coli*, enrofloxacin and ceftriaxone met this target. This was also the case for *Pasteurella multocida* for the antibiotics ampicillin and benzylpenicillin during meningitis and for chloramphenicol and enrofloxacin.

Overall, enrofloxacin met 11 of the 14 targets for which data was available, ampicillin during meningitis met 7 of the 12 targets for which data was available and benzylpenicillin during meningitis met 7 of the 10 targets for which data was available. All other antibiotics met no more than 4 targets. The values and concentrations utilized for the calculations described above are displayed in Tables 8–11. No relevant data about the susceptibility of the four different pathogens against sulfadiazine was available. Therefore, this analysis could not be performed for this substance. Data for cefotaxime was insufficient to perform this analysis.

In cats, this analysis was only possible for benzylpenicillin and clindamycin (Table 7). Penicillin showed a similar achievement of PK-PD targets as reported in dogs. Due to the lower MIC_90_ value of *Staphylococcus pseudintermedius* in cats vs. dogs, benzylpenicillin met the C_max_/MIC_90_ target for this pathogen in addition to the target met in dogs. Clindamycin met the C_max_/MIC_50_ targets for *Staphylococcus pseudintermedius* and *Staphylococcus aureus*, whereas other targets were not met. The values and concentrations utilized for the calculations described above are displayed in Table 12 for the investigated pathogens.

**Table 12:** C_max_ in CSF in cats after the administration of benzylpenicillin or clindamycin and selected PK-PD cutoffs of four bacterial pathogens.

**Antibiotic / Pathogen**	**C_**max**_ (μg/ml)**	**MIC_**50**_ (μg/ml)**	**MIC_**90**_ /(μg/ml)**	**PK-PD cutoff in μg/ml**	**Source of MIC**
Benzylpenicillin / *S. aureus*	1.5	0.5	8	0.75	32
Clindamycin / *S. aureus*	0.44	0.16	16	0.22	32
Benzylpenicillin / *Staph. pseudintermedius*	1.5	0.06	0.25	0.75	32
Clindamycin / *Staph. pseudintermedius*	0.44	0.06	16	0.22	32
Benzylpenicillin / *E. coli*	1.5	4	256	0.75	35
Clindamycin / *E. coli*	0.44	no data	no data	0.22	no data
Benzylpenicillin / *P. multocida*	1.5	0.12	0.12	0.75	32
Clindamycin / *P. multocida*	0.44	16	16	0.22	32

## Discussion

### Assessment of Potential Antimicrobial Effectiveness

The objective of this paper was to give clinicians an overview of the currently available pharmacokinetic data relevant for the use of antimicrobials in cases of bacterial infections of the central nervous system in dogs and cats. Furthermore, we attempted to make a preliminary assessment of the efficacy that can be expected when using these antimicrobials vs. different causative pathogens. To our knowledge, this is the first systematic review that attempts to address this specific set of problems.

The assessment of the efficacy of antimicrobials *in vivo* is classically based on different PK-PD indices and specific targets that should be met in order to enable a high chance of antimicrobial effectiveness (20), an approach known as PK-PD integration (78). The preferred PK-PD indices in veterinary medicine are %*f* T > MIC, which is defined as “the percentage of time during the dosing interval that plasma concentration of unbound drug exceeds MIC” (79), and *f* AUC/MIC, which is defined as “the ratio of the Area Under the Plasma Concentration-Time curve of free drug divided by MIC” (79). Both indices require information about multiple data points on the concentration time curve of an antimicrobial in the considered medium. Unfortunately, this information could not be extracted from the available literature. The only reliably available datapoint was the C_max_ in the CSF. Even though the PK-PD index C_max_/MIC is not the index of choice, its calculation may be of some value to the practitioner, especially in the absence of any other information on the possible effectiveness of antimicrobials against bacterial infections of the CNS that is not solely based on limited evidence like case reports or derived from theoretical considerations about the basic characteristics of antimicrobial substances. In our view, this is especially relevant for the decision about which antimicrobials approved for cats and dogs should not be chosen when attempting to treat such infections. If an antimicrobial is not able to achieve the required cutoff for the C_max_/MIC index with regards to the MIC_50_ and MIC_90_ and the ECOFF, it is unlikely that it can be of significant clinical benefit. An example for this would be chloramphenicol vs. *Staphylococcus aureus*. On the other hand, an antimicrobial that achieves the required cutoffs for this index with regards to the MIC_50_, the MIC_90_ and the ECOFF, has a substantial chance that at least some level of clinical efficacy exists. This is for example the case for ampicillin vs. *Pasteurella multocida* during meningitis. Judgements with a higher degree of certainty about the clinical efficacy of an antimicrobial would require more data than is currently available for this indication.

Even though few specific PK-PD cutoffs are available in veterinary medicine, like for doxycycline and tetracycline against *Staphylococcus pseudintermedius* in dogs (34), the application of PK-PD-targets reported in human medicine can be used to guide an approximate evaluation of the expected effectiveness of antibiotics in animals (79). The preferred PK-PD indices and the respective PK-PD targets are specific for different groups of antimicrobial substances. The typical PK-PD index for time dependent drugs like β- lactams, chloramphenicol and sulphonamides is %*f* T > MIC. This fraction should be > 50 % (80), but can also vary for example in different bacterial strains. (79). As no data for the concentration time curve of these substances in the CNS was available, a minimum C_max_ to target (MIC_50_, MIC_90_ or BP) ratio of 2 to 1 was used as a cut off value, which would in theory allow a dosing of the respective drug with an interval of every two half-live periods or longer if significant accumulation of the active substance occurs. For the time dependent antibiotic clindamycin, a PK-PD target of c > 2^*^MIC has been characterized in the literature (81). For tetracyclines, the preferred PK-PD index is the *f* AUC/MIC ratio, which should be above 25 h (34). This AUC/MIC ratio is roughly equivalent to a C_max_/MIC ratio of about 3:1 (82). For fluoroquinolones, an *f* AUC/MIC ratio > 125 h has been recognized as a valid PK-PD target, which typically corresponds to a C_max_/MIC ratio of about 10:1 (82). For aminoglycosides, a C_max_/MIC ratio of 8–10:1 has been proposed as an appropriate PK-PD target (68), even though AUC/MIC is today considered as the preferential PK-PD index (83). MICs of the different clinically relevant bacterial species are not static and may change over time. They can differ widely between different animal species, investigated sample material, pathologies and geographical origins (84). The choice of the medium in which an antimicrobial resistance test is performed can also have a large influence on the MIC. Furthermore, MICs are influenced by the used inoculum strength and their scientific validity is negatively impacted by the routine use of static concentrations of antimicrobials, which does not correctly reflect the variable concentrations of antimicrobials in patients (79).

Notably, clinical breakpoints, specific for a combination of antimicrobial agent, target bacteria, infected organ system and animal species, are missing (85). No specific MIC data for bacteria isolated from dogs and cats with bacterial meningitis are available. However, the source of most bacterial infections of the CNS in dogs and cats is considered to be a primary infection of other organs, like the ear and respiratory tract (2, 73). Therefore, MICs derived from bacterial pathogens isolated from patients that where diagnosed with the typical clinical signs of CNS infections, like cranial nerve deficits, ataxia, paresis, altered behavior and neck pain (2, 7), should exhibit characteristics similar to the pathogens involved in bacterial infections of the CNS in regard to their antimicrobial sensitivity.

In part, the analysis of numeric PK-PD-target threshold concentrations performed in this study provides evidence for the ability of different antimicrobial agents to reach effective CSF concentrations against selected bacteria which are often involved in meningitis in dogs and cats (Tables 6, 7), e.g., ampicillin against *Staphylococcus pseudintermedius* particularly during meningitis (Table 8). Because of the low protein concentration in the CSF of healthy animals, it is reasonable to assume that the total concentration of an antibiotic measured under these circumstances is roughly equal to its free concentration. This situation changes during meningitis, when the total protein concentration in the CSF rises (6) from below 0.5 g/l (86) to up to 7 g/l (87), which is equal to about 1/10^th^ of the reference value for the total plasma protein concentration in dogs. An elevated protein concentration in the CSF increases the total protein binding capacity of the CSF and can reduce the free concentration of antimicrobials. Unfortunately, quantifiable data for this is not available to our knowledge.

### Limitations

This systematic review reveals several limitations for recommendations concerning the choice of antibiotics. First, data on CSF concentrations are often completely missing, such as for metronidazole which is highly effective against anaerobic bacteria and recommended for the treatment of meningoencephalitis and brain abscesses (11, 88). While data on CSF concentrations was available for sulfadiazine, one of the sulphonamides which are known to penetrate the BBB, no current susceptibility data of relevant pathogens was available. Data was also lacking for trimethoprim. Thus, clinical recommendations for the use of metronidazole or trimethoprim/sulfadiazine cannot be evaluated from data in dogs and cats, although these agents are known to permeate the BBB and can reach sufficient brain concentrations in humans against different pathogens independently of inflammation of the meninges (11, 19). As a further limitation, many analyses of CSF concentrations were performed in the 1960s and 1970s, hence by outdated methods with lower accuracy than current standards like high performance liquid chromatography or mass spectroscopy. Furthermore, the number of animals investigated in these studies was often very small and most studies used a limited number of datapoints, in part only one time point (43), but a more conclusive evaluation of PK-PD -targets requires detailed information about time-concentration profiles of the investigated drugs (89). In addition, data on protein binding of antibiotics in CSF, important for the assessment of their efficacy *in vivo* (90), are not available in dogs and cats. This is less relevant in healthy animals where the protein concentration in the CSF is low and a significant protein binding is unlikely to occur. In contrast, the protein concentration in the CSF of animals with infectious is higher and can have a relevant effect on the unbound fraction of the respective substance. Most studies did not investigate the pharmacokinetics of the respective antibiotic after repeated administration which usually leads to higher concentrations than a single administration if proper dosage intervals are observed. As mentioned above, the PK-PD index C_max_/MIC is not the index of choice and therefore offers only a reduced informative value.

### Treatment Options and Recommendations

Bacterial meningitis in cats and dogs can be caused by a variety of species. Frequently reported causative bacteria include the Gram-positive species *Staphylococcus* spp. (91) and *Streptococcus* spp. (2, 92) and the Gram-negative species *Pasteurella multocida* (92), *E. coli* and *Klebsiella* spp. (2). Furthermore, anaerobic bacteria like Gram-negative *Bacteroides* spp.*, Fusobacterium* spp. and *Prevotella oralis* (5) and Gram-positive *Clostridium* spp. can be involved (2). A successful identification of the involved pathogens by microbiological analysis can support the rational choice of antibiotics, but cultures from patients with bacterial meningitis may only be positive in a variable number of patients ranging from 13% in one study of dogs (2) to up to 85% in humans (8). Since antibacterial treatment of CNS infections should be initiated as soon as possible and co-infections with multiple bacterial species occur in a significant proportion of affected animals (2, 5), empirical treatment with broad spectrum antibiotics or combinations of compounds are rational therapeutic options.

Recommendations for the treatment of bacterial infections of the central nervous system in cats and dogs are often derived from case reports, studies with small sample sizes as well as extrapolated from human medicine (11). Generally accepted clinical guidelines for empirical antimicrobial therapy of bacterial meningitis and brain abscesses in humans (93) include benzylpenicillin, broad spectrum beta-lactam antibiotics like the aminopenicillins ampicillin and amoxicillin and the third-generation cephalosporines ceftriaxone and cefotaxime. Fluoroquinolones are usually not recommended as first line therapy but are mentioned as alternatives or in combination especially because of a high activity against Gram-negative bacteria. Metronidazole, which achieves an AUC CSF/*f* AUC plasma ratio of about 0.86 in humans (94) is recommended against anaerobic bacteria (95). Other permeable antibiotics, such as chloramphenicol, are regarded as alternatives for patients with allergy to beta-lactam antibiotics or with CNS infections caused by multi-resistant pathogens (19). In contrast, a generally accepted therapeutic protocol could not be identified for the empirical treatment of CNS infections in dogs and cats. However, several case reports indicate an frequent selection of broad spectrum β-lactams, fluoroquinolones, trimethoprim-sulfadiazine, metronidazole and clindamycin for CNS infections of dogs and cats with varying degrees of success (1, 70, 96). In addition to the selection of an effective antibiotic, the question of optimal dosing has to be considered. Table 13 compares the approved dosages and the dosages recommended in the literature of antimicrobial agents used for the treatment of bacterial infections of the CNS in dogs and cats. The dosages recommended for the treatment of bacterial CNS infections are usually higher than the dosages used for the treatment of peripheral infections. As highlighted above, very little data about the pharmacokinetic properties of the respective antibiotics are available. Therefore, most dosages recommended in the literature are not based on a sufficient level of evidence and should be regarded as empirical recommendations. Nevertheless, the general principle of most recommendations to use high dosages, preferentially *via* parenteral modes of application, is reasonable as this increases the likelihood to achieve the required PK-PD index cutoff in the CSF (19). An exceedance of the approved dose of an antibiotic can increase the likelihood of adverse events. This is especially relevant for antimicrobials which are associated with central adverse effects when used in high dosages, which has been described in dogs and cats for metronidazole (97, 98), chloramphenicol (99) and procaine benzylpenicillin (100). High dosages of fluoroquinolones have been associated with an elevated risk of seizures especially in epileptic dogs (101) and retinal degeneration in cats (102). Therefore, any increase of the administered dose above the approved dose of a given antibiotic should only be performed after a careful consideration of the associated risks and benefits.

**Table 13:** Approved and recommended doses of antibacterial substances for empirical treatment of CNS infections and peripheral infections in dogs (for comparisons of doses used in PK studies see Tables 4, 5).

**Active Substance**	**Approved dose for various peripheral infections (trade name given as example)**	**Dose recommended for the treatment of CNS infections (example of reference)**
Ampicillin	12–24 mg/kg i.v. q6h-q8h (Ampi-Dry®)	40 mg/kg i.v. q6h (69)
Amoxicillin	10 mg/kg i.m. q12h (Amoxicillin 15% WDT®)	50 mg/kg i.v. every q6h (70)
Benzylpenicillin	10.000–20.000 U/kg i.m. q4h-q6h (Penicillin-G-Natrium®)	10.000–22.000 U/kg i.v. q4h-q6h (71)
Cefotaxime	Not approved for animals in EU	20–50 mg/kg IV q8h (11)
Ceftriaxone	Not approved for animals in EU	50 mg/kg s.c / i.m. q12h-q24h (all susceptible infections) (72)
Chloramphenicol	30 mg/kg p.o. q12h (Chloro-Sleecol®)	50 mg/kg p.o. q8h (56)
Clindamycin (incl. cats)	5.5 mg/kg p.o. q12hy or 11 mg/kg p.o. q24h (Zodon®)	20 mg/kg i.v. q12h for 2 d followed by 11 mg/kg p.o. q24h in cats (73)
Doxycycline	10 mg/kg p.o. q12h (Ronaxan®)	5 mg/kg p.o. q12h (74)
Enrofloxacin	5 mg/kg s.c. q24h (Baytril®)	5–10 mg/kg p.o. q12h (71)
Metronidazole	25 mg/kg p.o. q12h (Eradia®)	10–15 mg/kg q8h (71) 20 mg/kg i.v. q12h (75)
Pradofloxacin	3–4.5 mg/kg p.o. q24h (Veraflox®)	3–6 mg/kg p.o. q24h (50)
Trimethoprim-Sulphonamides (incl. cats)	15 mg/kg p.o. q12h (TSO-Tabletten®)	15–30 mg/kg p.o. q12h (76, 77)

## Conclusions

The state of the pharmacological evidence for the treatment of bacterial infections of the CNS in dogs and cats is incomplete and insufficient for definitive therapeutic recommendations. While still fragmentary on an absolute basis, the best evidence for their effectiveness against bacterial meningitis relative to all other antimicrobials approved for dogs exists for enrofloxacin, benzylpenicillin and ampicillin. A reduced level of supporting evidence exists for the effectiveness of ceftriaxone and to a somewhat lesser extend for the effectiveness of cefoxitin, two substances not approved in veterinary medicine. No data could be found for metronidazole and trimethoprim-sulphonamides, which does not preclude their potential effectiveness in bacterial meningitis. The evidence in cats is particularly deficient, as data could only be retrieved for two active substances.

To improve the cure rate of dogs and cats afflicted by bacterial meningitis, studies investigating the pharmacokinetic properties of promising antimicrobials should be performed. This would allow more conclusive statements about the optimal dosage regimen and drug selection for the treatment of this disease.

## Data Availability Statement

The original contributions presented in the study are included in the article/supplementary material, further inquiries can be directed to the corresponding author.

## Author Contributions

AR and RH: conceived the idea and drafted and edited the manuscript. RH: performed literature search and extracted relevant data. All authors contributed to the article and approved the submitted version.

## Conflict of Interest

The authors declare that the research was conducted in the absence of any commercial or financial relationships that could be construed as a potential conflict of interest.

## Publisher's Note

All claims expressed in this article are solely those of the authors and do not necessarily represent those of their affiliated organizations, or those of the publisher, the editors and the reviewers. Any product that may be evaluated in this article, or claim that may be made by its manufacturer, is not guaranteed or endorsed by the publisher.

## Abbreviations

AUC: area under the curve
BBB: blood brain barrier
BCSFB: blood cerebrospinal fluid barrier
BP: breakpoint
CNS: central nervous system
CSF: cerebrospinal fluid
fAUC: area under the curve of the free drug
fCmax: maximum concentration of the free drug
fu: free plasma fraction
Cmax: maximum concentration
MIC: minimum inhibitory concentration
PK-PD: pharmacokinetic–pharmacodynamic
VMP: veterinary medicinal product.
